# Resveratrol prevents sarcopenic obesity by reversing mitochondrial dysfunction and oxidative stress via the PKA/LKB1/AMPK pathway

**DOI:** 10.18632/aging.101910

**Published:** 2019-04-15

**Authors:** Yujie Huang, Xiaohui Zhu, Ka Chen, Hedong Lang, Yong Zhang, Pengfei Hou, Li Ran, Min Zhou, Jiawei Zheng, Long Yi, Mantian Mi, Qianyong Zhang

**Affiliations:** 1 Research Center for Nutrition and Food Safety, Chongqing Key Laboratory of Nutrition and Food Safety, Institute of Military Preventive Medicine, Third Military Medical University, Shapingba District, Chongqing 400038, P. R. China

**Keywords:** sarcopenic obesity, resveratrol, mitochondrial function, oxidative stress, AMPK

## Abstract

Background: The concept of sarcopenic obesity refers to low muscle mass coupled with high adiposity in older adults. Sarcopenic obesity is a new medical challenge that imposes tremendous financial burdens on healthcare authorities worldwide. This study investigated the effects of resveratrol on high-fat diet-induced sarcopenic obesity in aged rats and palmitate acid-induced muscle atrophy in L6 myotubes and explored the underlying mechanisms.

Results: *In vivo*, resveratrol prevented muscle loss and myofiber size decrease, improved grip strength and abolished excessive fat accumulation. *In vitro*, resveratrol inhibited the palmitate acid-mediated reductions in myosin heavy chain content and myotube diameter. Moreover, resveratrol ameliorated mitochondrial dysfunction and oxidative stress, leading to an improvement in protein metabolism and contributing to the prevention of muscle atrophy. Furthermore, the protective effects of resveratrol on mitochondrial function, oxidative stress and muscle atrophy were abolished by PKA siRNA, LKB1 siRNA and AMPK siRNA transfection *in vitro*.

Conclusions: Resveratrol prevented high-fat diet-induced muscle atrophy in aged rats by reversing mitochondrial dysfunction and oxidative stress, which was partially mediated by the PKA/LKB1/AMPK pathway. These findings indicate that resveratrol might have potential uses for the prevention and treatment of sarcopenic obesity.

## Introduction

Sarcopenia, the age-related loss of skeletal muscle mass and strength, is closely associated with physical functional limitations, frailty and poor quality of life [1–3]. Accumulating evidence suggests that obesity and excessive intake of saturated fatty acids can exacerbate sarcopenia [4–6], leading to a higher risk of metabolic disorders and increased morbidity and mortality [7]. The combination of reduced muscle mass and increased body fat in older adults, which was recently defined as sarcopenic obesity, represents a serious public health concern, and its prevalence is increasing throughout the world [8]. Hence, effective and safe strategies for the prevention and therapy of sarcopenic obesity are urgently needed to promote healthy aging and extend life expectancy.

In the past several years, a growing body of literature has highlighted the pivotal role of mitochondrial dysfunction and oxidative stress in the pathogenesis of muscle atrophy during aging [9–12]. Moreover, mitochondrial dysfunction and oxidative stress are considered contributors to obesity or fatty acid-induced muscle atrophy [6,13]. Thus, identification of the molecular mechanisms regulating mitochondrial function and oxidative stress, as well as the development of strategies that could alleviate mitochondrial dysfunction and oxidative stress, might be beneficial for the management of sarcopenic obesity.

AMP-activated protein kinase (AMPK), a sensor of the cellular energy status, acts as a key regulator of mitochondrial function and oxidative stress in skeletal muscle [14–16]. Moreover, previous investigations have demonstrated that AMPK activity is reduced in skeletal muscle tissues of high-fat diet (HFD)-fed rats [17,18]. Liver kinase B1 (LKB1), a major upstream AMPK kinase, has been shown to promote mitochondrial biogenesis and antioxidative pathways in skeletal muscle after its activation [19–21]. Protein kinase A (PKA), an upstream kinase in the LKB1/AMPK pathway [22], has been identified as a potential target for muscle wasting in several conditions [23]. These findings suggest that targeting the PKA/LKB1/AMPK signaling pathway might constitute a plausible approach for enhancing mitochondrial function and blocking oxidative stress in sarcopenic obesity.

Resveratrol (RSV), a natural polyphenol present in many plant species, exerts a broad spectrum of health benefits, including antioxidative, anti-inflammatory, antitumor, and antiobesity properties [24]. In recent years, the antiaging effect of RSV has aroused considerable interest worldwide [25]. Because skeletal muscle plays key roles in the systemic regulation of aging and age-related diseases [26], the effects of RSV on the lifespan and overall aging of an organism might be closely associated with the effects of the polyphenol on skeletal muscle. RSV has been reported to prevent muscle atrophy in several catabolic conditions, including cancer, diabetes, chronic kidney disease and disuse [27–30]. Moreover, previous studies have demonstrated that RSV ameliorates aging-induced oxidative damage in skeletal muscle [31], and we previously found that RSV prevents HFD-induced hepatic steatosis through activation of the PKA/AMPK signaling pathway [32]. These results led us to hypothesize that RSV might protect against sarcopenic obesity by attenuating mitochondrial dysfunction and oxidative stress through the PKA/LKB1/AMPK pathway. To verify this hypothesis, we evaluated the effects of RSV on HFD-induced sarcopenic obesity in aged rats and palmitate acid (PA)-induced muscle atrophy in myotubes. The changes in mitochondrial function and oxidative stress, as well as the potential involvement of the PKA/LKB1/AMPK signaling pathway, were also investigated.

## RESULTS

### RSV protected against HFD-induced muscle atrophy and dysfunction in aged rats

**Figure 1:**
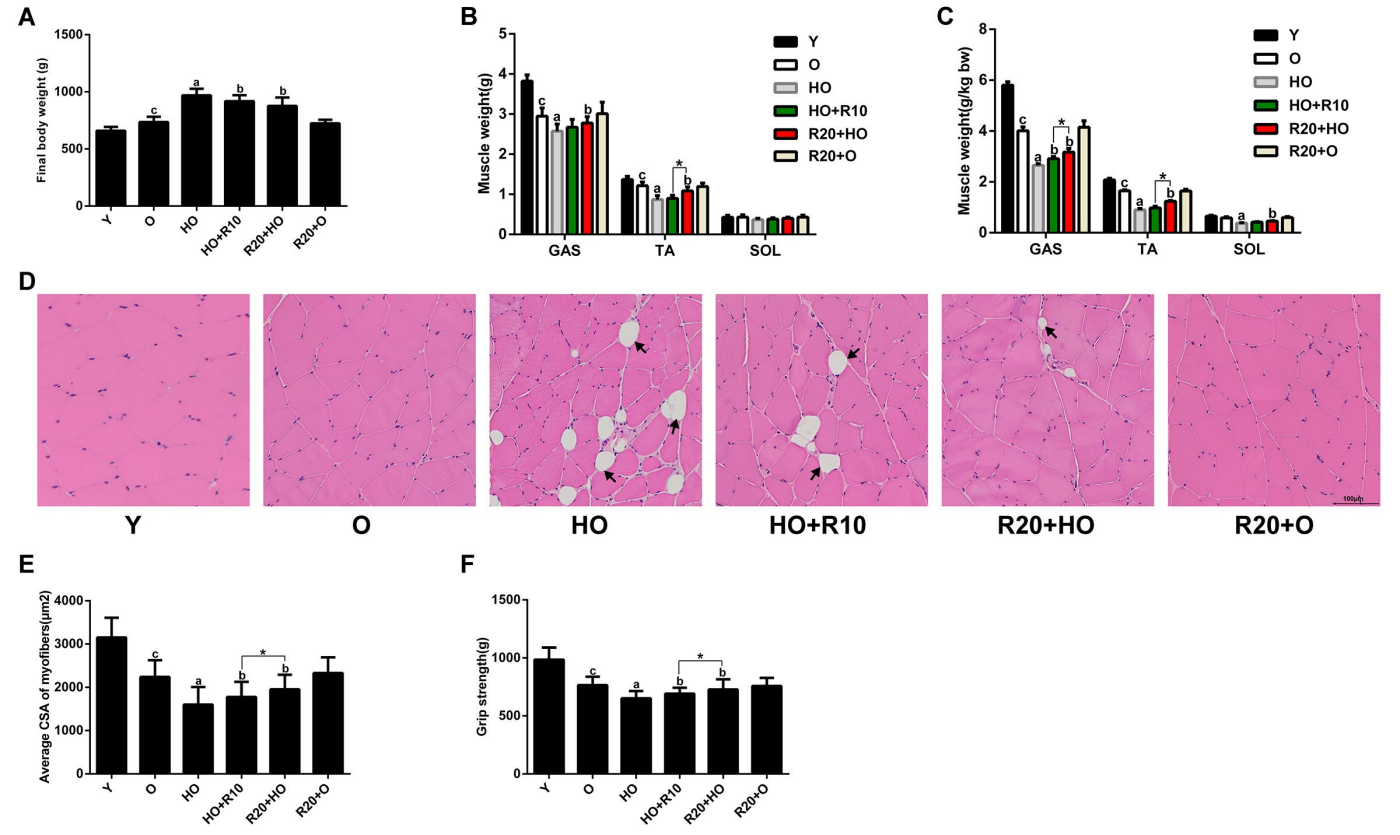
**RSV protects against HFD-induced muscle atrophy in aged rats.** (**A**) Final body weights of the different groups. (**B**) Weights of GAS, TA and SOL muscles in each group. (**C**) The relative muscle weights in each group. (**D**) Representative images of H&E staining of the GAS muscles. Scale bar, 100 μm. (**E**) Average muscle fiber CSA of the GAS muscles. (**F**) Grip strength in each group. Data are expressed as the mean ± SD. ^a^*P* < 0.05 vs. the O-group; ^b^*P* < 0.05 vs. the HO-group; ^c^*P* < 0.05 vs. the Y-group; *P < 0.05 for the comparison between the marked groups.

Six groups of rats were formed: young rats fed the normal chow diet (CD) (Y), old rats fed the normal CD (O), old rats fed the HFD (HO), old rats fed the HFD for 10 weeks and then the HFD supplemented with RSV for 10 weeks (HO+R10), old rats fed the HFD supplemented with RSV starting from the time of study initiation and throughout the 20-week experimental period (R20+HO), and old rats fed a normal CD supplemented with RSV starting from the time of study initiation and throughout the 20-week experimental period (R20+O). Prior to the experimental period, no apparent difference in baseline body weight was observed between the groups of aged rats (Figure S1A). After 20 weeks of feeding, the HFD-fed aged rats exhibited a higher body weight than the CD-fed aged rats (Figure 1A). The weights of the gastrocnemius (GAS) and tibialis anterior (TA) muscles were reduced during aging and further decreased in the aged rats fed the HFD (Figure 1B and 1C). Although the absolute weights of the soleus (SOL) muscles showed no overt differences among the six groups, the relative SOL muscle weights of the HO group were lower than those of the O group (Figure 1B and 1C). RSV supplementation reversed the HFD-induced increase in body weight and decreases in muscle weights (Figure 1A-C). A histological analysis showed that the mean fiber cross-sectional area (CSA) of the GAS muscles was decreased during aging, and a greater reduction was observed in the HFD-fed aged rats. Moreover, the rats in the HO group developed a large number of lipid droplets in their GAS muscles. The addition of RSV to the HFD attenuated these histological changes (Figure 1D and 1E). To monitor muscle function, we conducted a grip strength test. Concomitant with the changes in muscle weights, the grip strength was decreased by aging and further decreased in the aged rats fed the HFD. RSV administration prevented the HFD-induced reduction in grip strength (Figure 1F). Moreover, the weights and fiber CSA of the GAS muscles and grip strength were higher in the R20+HO group than in the HO+R10 group, and no significant differences in body weight, muscle weights, muscle fiber CSA and grip strength were observed between the O group and the R20+O groups (Figure 1). In addition, the addition of RSV to the diet increased the serum concentrations of RSV in aged rats (Figure S1B). Together, these results suggested that RSV supplementation effectively protected against HFD-induced muscle wasting and dysfunction in aged rats.

### RSV inhibited fat accumulation and ameliorated adverse changes in serum biochemical parameters

**Figure 2:**
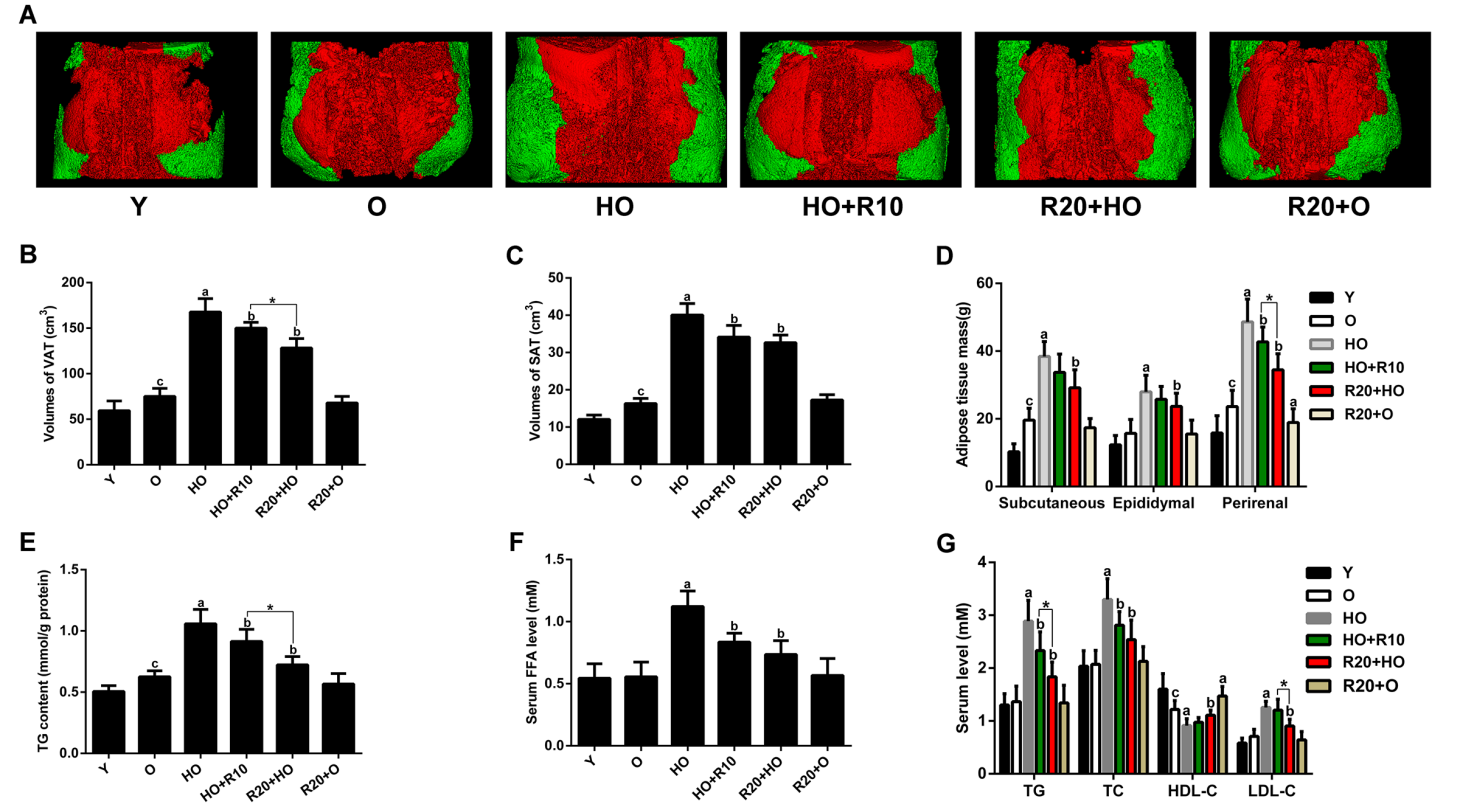
**Effects of RSV on adipose tissue volumes and masses, intramuscular TG content and serum lipid profile.** (**A**) Representative three-dimensional images of abdominal fat tissues (red, visceral fat; green, subcutaneous fat). (**B**) VAT volumes. (**C**) SAT volumes. (**D**) Fat masses. (**E**) TG content of the GAS muscles. (**F**) Serum FFA levels. (**G**) Serum TG, TC, HDL-C and LDL-C levels. Data are expressed as the mean ± SD. ^a^*P* < 0.05 vs. the O group; ^b^*P* < 0.05 vs. the HO group; ^c^*P* < 0.05 vs. the Y group; *P < 0.05 for the comparison between the marked groups.

The abdominal fat tissues were analyzed through micro-computed tomography (microCT). As shown in Figure 2A-C, the volumes of visceral adipose tissue (VAT) and subcutaneous adipose tissue (SAT) were increased during aging and further increased in aged rats fed the HFD. Paralleling the changes in the fat mass volumes, the weights of subcutaneous, epididymal and perirenal fat tissues were elevated by HFD feeding (Figure 2D). We also measured the TG levels in the GAS muscles and found that the intramuscular fat content was greater with aging and further elevated in the HFD-fed aged rats (Figure 2E). The serum levels of free fatty acid (FFA), total triglyceride (TG), total cholesterol (TC) and low-density lipoprotein cholesterol (LDL-C) of the HO group were higher than those of the O group, and these increases were concomitant with a decrease in the serum high-density lipoprotein cholesterol (HDL-C) levels (Figure 2F and 2G). RSV supplementation significantly reduced the fat masses and ameliorated the adverse changes in the serum lipid levels observed in the HFD-fed aged rats (Figure 2). Moreover, the VAT volumes, perirenal adipose tissue weights, intramuscular TG content, and serum TG and LDL-C levels were lower in the R20+HO group than in the HO+R10 group. In addition, RSV administration reduced the weights of perirenal adipose tissues and increased the serum HDL-C levels in aged rats fed the CD (Figure 2D and 2G).

### RSV ameliorated the HFD-induced mitochondrial morphological abnormalities in aged rats

**Figure 3:**
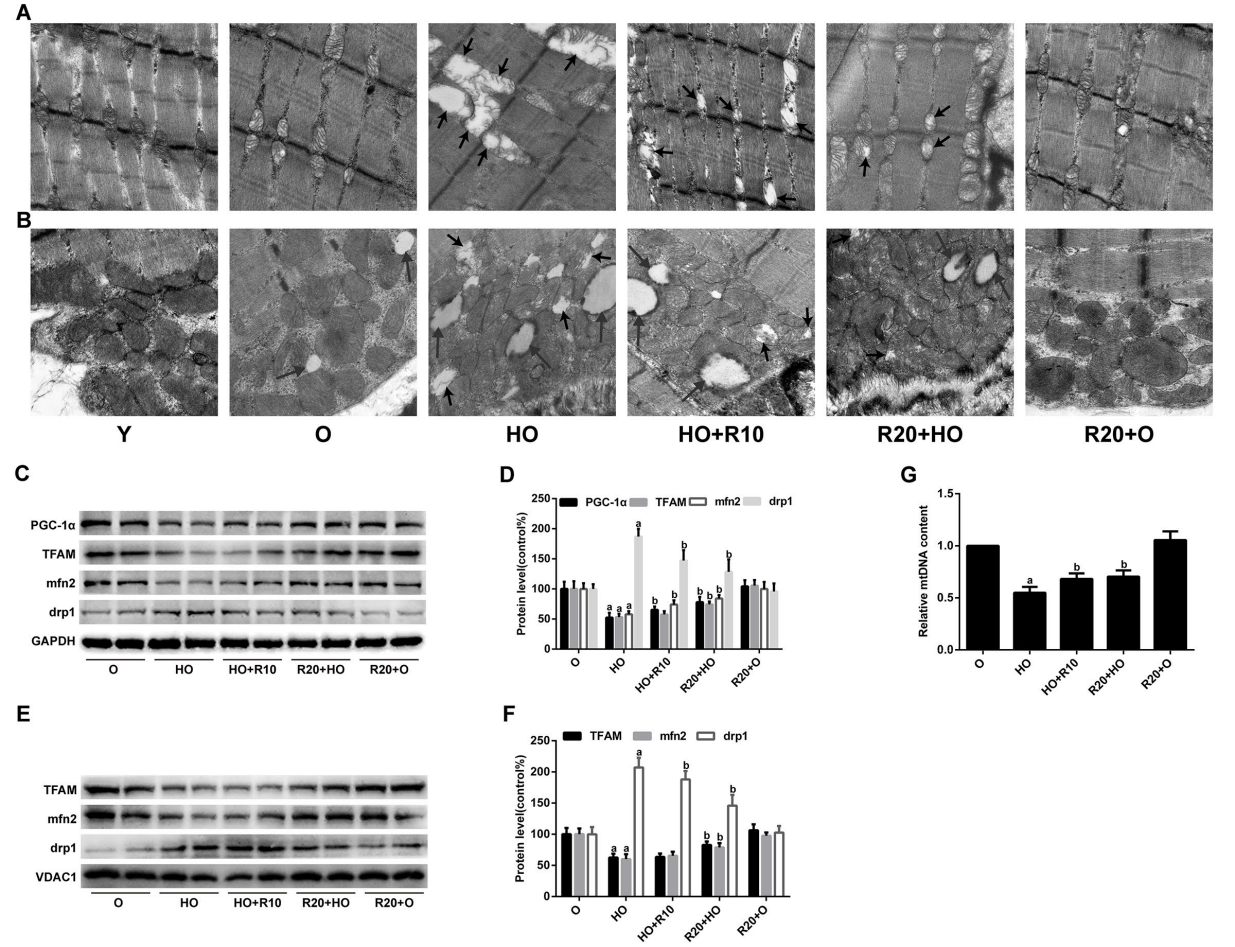
**Effects of RSV on mitochondrial morphology and related protein expression in the GAS muscles.** (**A** and **B**) Representative TEM images of the IFM and SSM at an original magnification of 25,000×. The short arrows indicate damaged mitochondria; the long arrows indicate lipid droplets. (**C**) Representative images of the western blotting results for PGC-1α, TFAM, mfn2 and drp1 in whole muscle lysates; GAPDH was used as a loading control. (**D**) The bar graphs show quantification of the indicated proteins. (**E**) Representative images of the western blotting results for TFAM, mfn2 and drp1 in the mitochondrial subfractions; VDAC1 was used as a loading control. (**F**) The bar graphs show quantification of the indicated proteins. (**G**) Relative mtDNA content. Data are expressed as the mean ± SD. ^a^*P* < 0.05 vs. the O group; ^b^*P* < 0.05 vs. the HO group.

We subsequently analyzed the mitochondrial morphology changes in the intermyofibrillar mitochondria (IFM) and subsarcolemmal mitochondria (SSM) through transmission electron microscopy (TEM). The images revealed fewer mitochondrial cristae and the presence of some lipid droplets closely adherent to SSM during aging, and these alterations were more extreme in the HFD-fed aged rats. Moreover, the aged rats fed the HFD showed many swollen mitochondria with distorted cristae and broken membranes (Figure 3A and 3B). The addition of RSV to the HFD ameliorated these morphological abnormalities, resulting in decreased amounts of aberrant mitochondria and smaller lipid droplets (Figure 3A and 3B).

We further evaluated the protein expression of key regulators involved in mitochondrial biogenesis and dynamics. As shown in Figure 3C and 3D, the decreases in the protein levels of PGC-1α, TFAM and mfn2, as well as the increased drp1 expression in HFD-fed aged rats, were all reversed by RSV supplementation. Similar alterations were observed for the protein levels of TFAM, mfn2 and drp1 in the mitochondrial subfractions (Figure 3E and 3F). In addition, RSV administration inhibited the HFD-mediated reductions in mtDNA copy number (Figure 3G).

### RSV improved mitochondrial function and antioxidant capacity in aged rats

**Figure 4:**
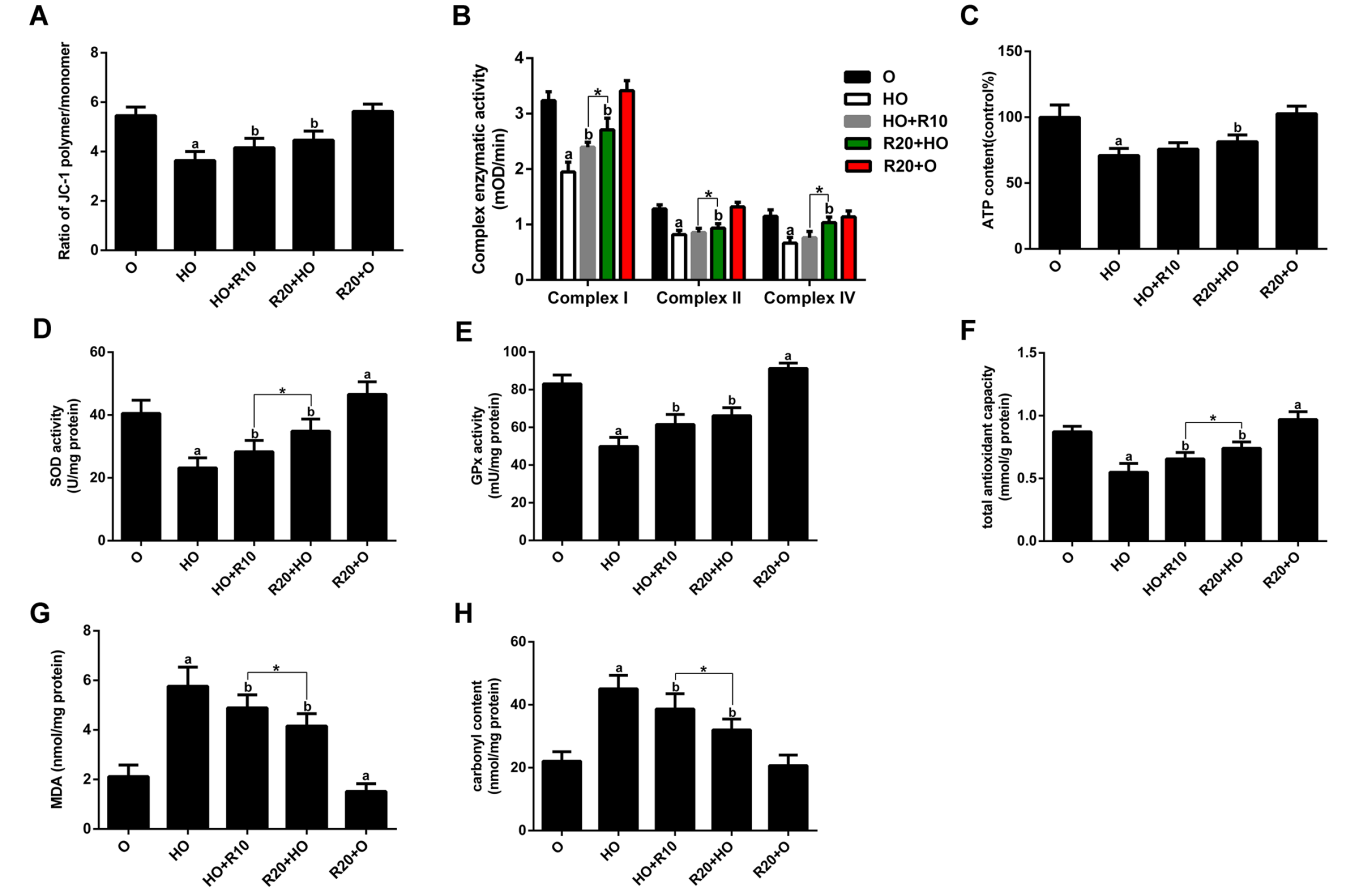
**Effects of RSV on mitochondrial function and oxidative stress in the GAS muscles.** (**A**) Δψm. (**B**) Activities of complexes I, II, and IV. (**C**) ATP content. (**D** and **E**) Activities of SOD and GPx. (**F**) T-AOC. (**G**) MDA levels. (**H**) Carbonyl protein content. Data are expressed as the mean ± SD. ^a^*P* < 0.05 vs. the O group; ^b^*P* < 0.05 vs. the HO group; *P < 0.05 for the comparison between the marked groups.

To investigate the effects of RSV on mitochondrial function, we analyzed the mitochondrial membrane potential (Δψm), mitochondrial respiratory chain complex activities and ATP production in the GAS muscles. The data showed that RSV supplementation prevented the HFD-induced loss of Δψm (Figure 4A). Furthermore, the activities of complexes I, II, and IV and the ATP content were decreased by HFD feeding but restored by RSV administration (Figure 4B and 4C).

To examine the effects of RSV on oxidative status, we measured the antioxidant enzyme activities and the levels of several oxidative damage markers in skeletal muscle tissues. We found that HFD feeding resulted in decreased total antioxidative capability **(**T-AOC) and reduced activity of superoxide dismutase (SOD) and glutathione peroxidase (GPx) accompanied by elevated malondialdehyde (MDA) and carbonyl protein contents. These changes were reversed by RSV supplementation (Figure 4D-F). Moreover, the T-AOC and activities of complexes I, II, and IV and superoxide dismutase (SOD) of the R20+HO group were higher than those of the HO+R10 group, and the MDA and carbonyl protein levels of the R20+HO group were lower than those of the HO+R10 group. In addition, RSV administration enhanced the antioxidant capacity and decreased the MDA levels in aged rats fed the CD (Figure 4D-H).

### RSV prevented PA-induced muscle atrophy *in vitro*

**Figure 5:**
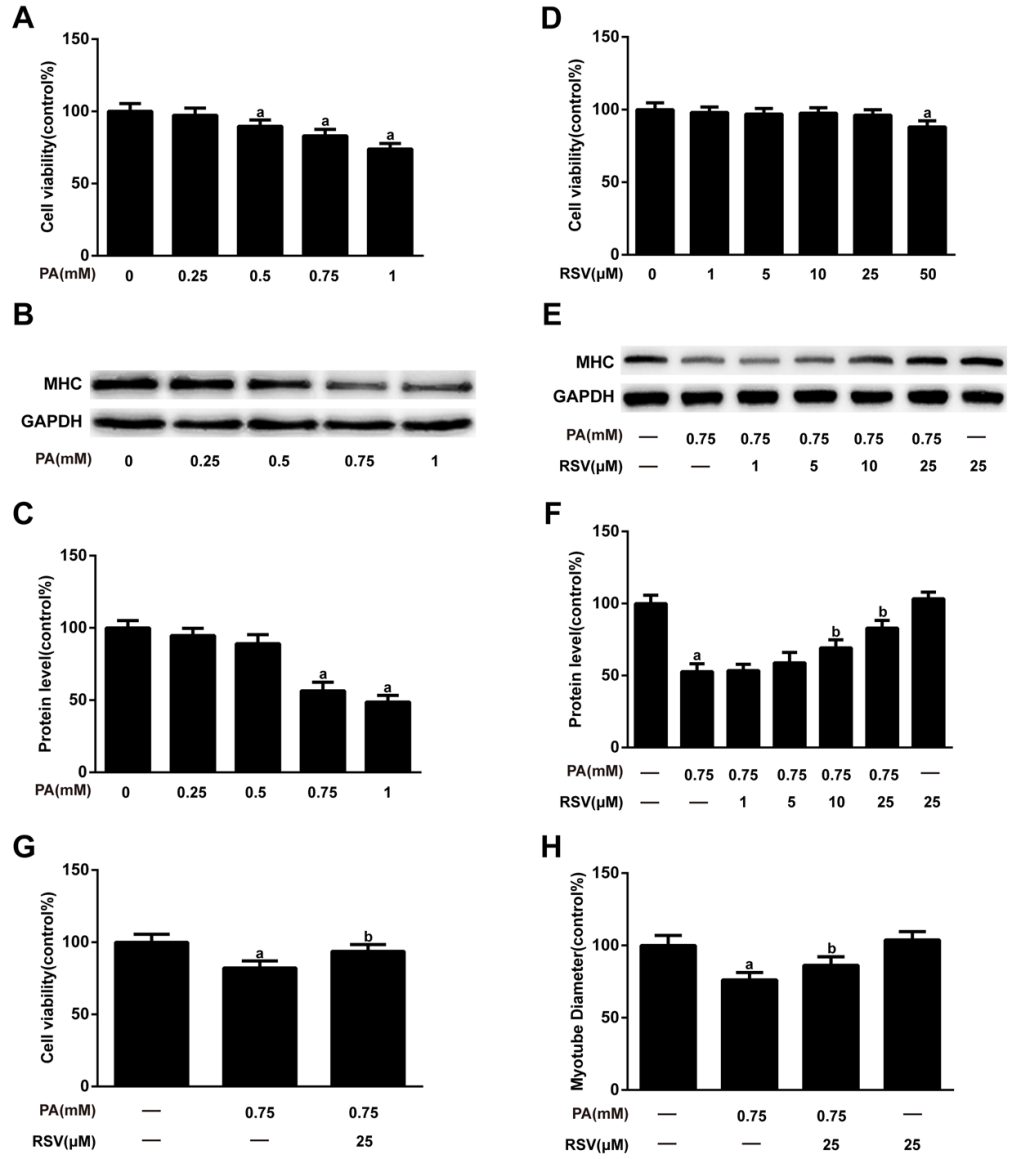
**RSV inhibits PA-induced muscle atrophy in L6 myotubes.** (**A**) Myotubes were treated with different doses (0.25, 0.5, 0.75, or 1 mM) of PA for 24 h, and cell viability was measured using a CCK-8 assay. (**B**) Representative image of the western blotting results for MHC; GAPDH was used as a loading control. (**C**) The bar chart shows the quantification of MHC. (**D**) Myotubes were treated with RSV at a series of concentrations (1, 5, 10, 25 or 50 μM) for 24 h, and cell viability was detected by CCK-8 assay. (**E**) Myotubes were treated with 0.75 mM PA in the presence or absence of different concentrations (1, 5, 10 and 25 μM) of RSV for 24 h. The protein expression of MHC was measured by western blotting. (**F**) The bar graph shows the quantification of MHC. (**G**) Myotubes were exposed to 0.75 mM PA in the presence or absence of 25 μM RSV for 24 h, and cell viability was detected by a CCK-8 assay. (**H**) Quantification of the myotube diameter, as described in the Materials and Methods section. Data are expressed as the mean ± SD. ^a^*P* < 0.05 vs. the control group; ^b^*P* < 0.05 vs. the PA-treated group.

To mimic HFD-induced muscle atrophy in aged rats, we used PA, the most abundant circulating saturated fatty acid [4], to produce a cell model of muscle atrophy. As shown in Figure 5A, no significant changes in cell viability were observed in myotubes treated with PA at concentrations of less than 0.5 mM. In addition, 0.75 mM PA notably reduced the myosin heavy chain (MHC) expression in myotubes (Figure 5B and 5C), and this concentration was therefore selected for the *in vitro* induction of the muscle atrophy model in subsequent experiments. Next, cells were treated with different concentrations of RSV to test its *in vitro* effects. RSV (1, 5, 10 and 25 μM) itself had no significant effects on cell viability in myotubes (Figure 5D) and dose-dependently inhibited the PA-induced decrease in MHC content (Figure 5E and 5F). RSV at the dose of 25 μM exerted the greatest protective effects; therefore, we chose this dose in subsequent experiments. Furthermore, the decreases in cell viability and myotube diameter, as well as the increased TG content in PA-treated myotubes, were all attenuated by RSV (Figure 5G, 5H and S2A). In addition, RSV alone exerted no significant effects on MHC expression, myotube diameter or TG content (Fig 5E, 5F, 5H and S2A). Together, these data indicated that RSV efficiently alleviated PA-induced cell death and muscle atrophy *in vitro*.

### RSV activated the PKA/LKB1/AMPK signaling pathway

**Figure 6:**
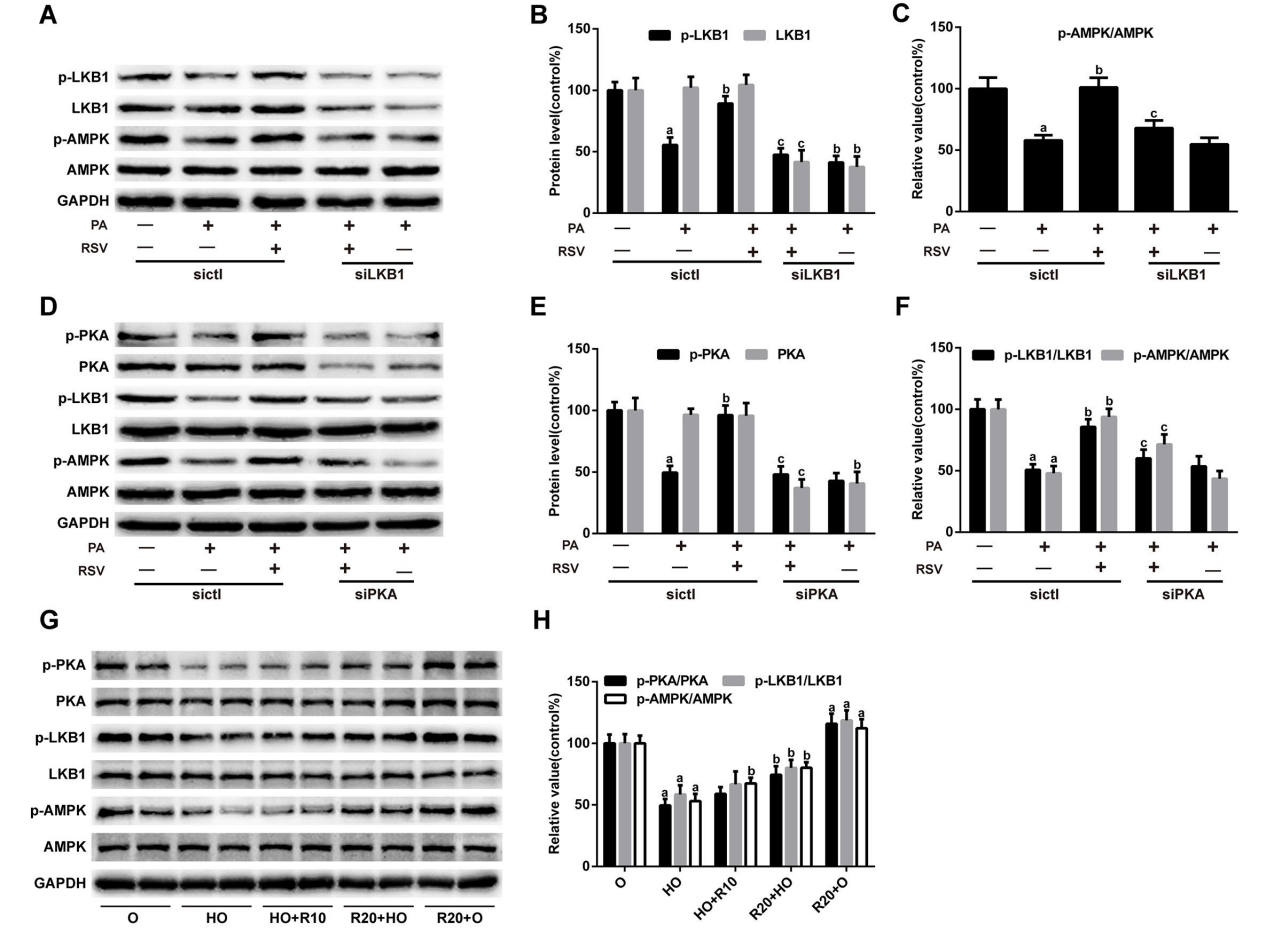
**RSV activates the PKA/LKB1/AMPK signaling pathway.** (**A**) Representative images of the western blotting results for p-LKB1, LKB1, p-AMPK and AMPK in myotubes; GAPDH was used as a loading control. (**B**) The bar charts show quantification of the indicated proteins. (**C**) Quantitative graph shows the ratios of p-AMPK/AMPK. (**D**) Representative images of the western blotting results for p-PKA, PKA, p-LKB1, LKB1, p-AMPK and AMPK in myotubes; GAPDH was used as a loading control. (**E**) The bar charts show quantification of the indicated proteins. (**F**) Quantitative graph shows the ratios of p-LKB1/LKB1 and p-AMPK/AMPK. Data are expressed as the mean ± SD. ^a^*P* < 0.05 vs. the control group; ^b^*P* < 0.05 vs. the PA-treated group; ^c^*P* < 0.05 vs. the RSV (25 μM) and PA (0.75 mM) cotreated group with control siRNA transfection. (**G**) Representative images of the western blotting results for p-PKA, PKA, p-LKB1, LKB1, p-AMPK and AMPK in the GAS muscles; GAPDH was used as a loading control. (**H**) The bar charts show the relative protein levels. Data are expressed as the mean ± SD. ^a^*P* < 0.05 vs. the O group; ^b^*P* < 0.05 vs. the HO group.

We further explored the possible mechanism underlying the protective effects of RSV in muscle atrophy models. As shown in Figure 6A-F, RSV restored the PA-mediated reductions in p-PKA, p-LKB1 and p-AMPK protein levels. Moreover, LKB1 siRNA inhibited RSV-induced LKB1 activation accompanied by decreased expression of p-AMPK (Figure 6A and 6C). Furthermore, PKA siRNA abolished the effects of RSV on the p-LKB1 and p-AMPK levels (Figure 6D and 6F). RSV supplementation also increased the expression of p-PKA, p-LKB1 and p-AMPK in the GAS muscles of HFD-fed rats (Figure 6G and 6H). These results suggested that RSV activated the PKA/LKB1/AMPK signaling pathway in the HFD- and PA-induced muscle atrophy models.

### RSV improved mitochondrial morphology, inhibited mtDNA depletion and restored mitochondrial-related protein expression in PA-treated L6 myotubes

**Figure 7:**
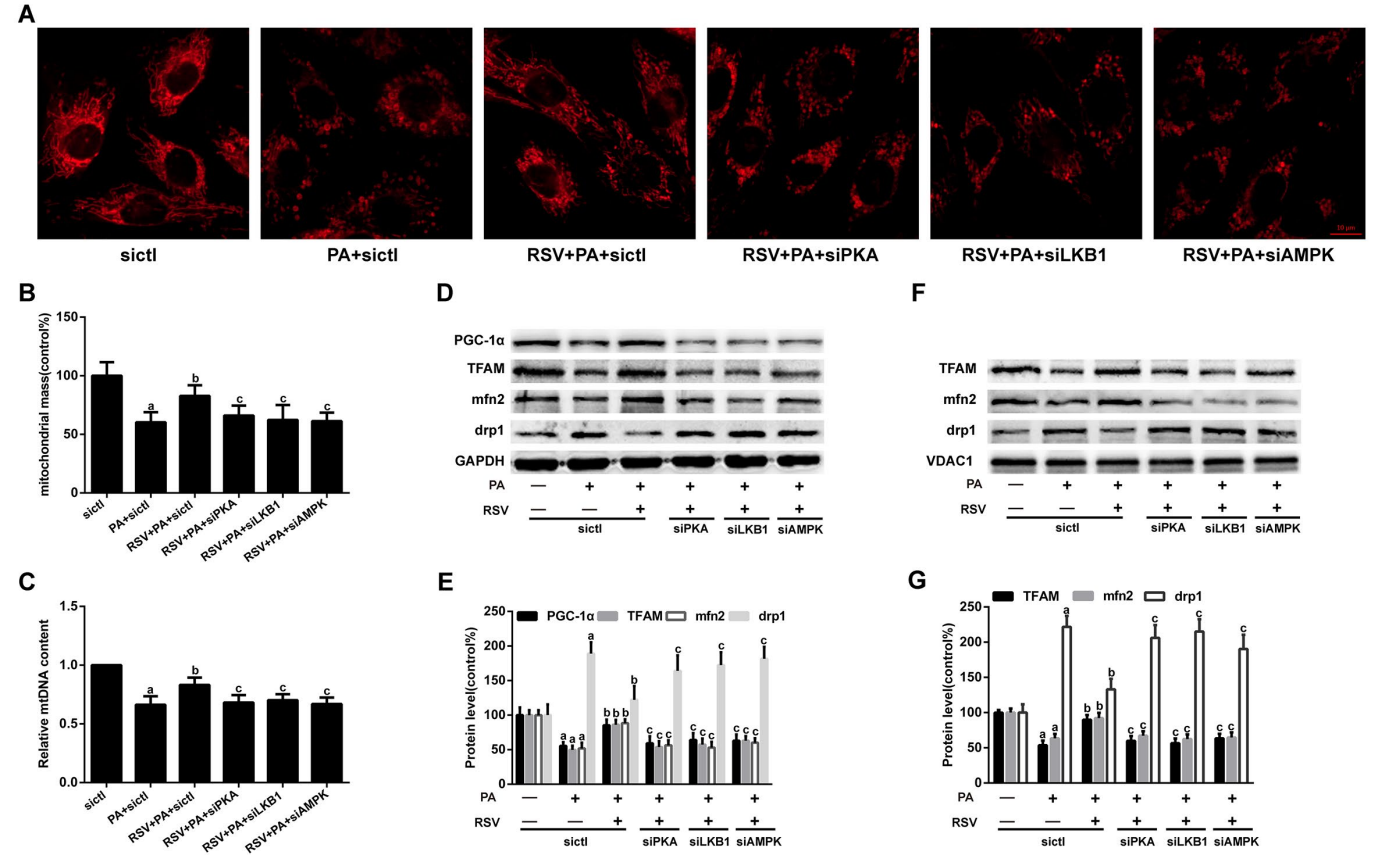
**Effects of RSV on mitochondrial morphology, mtDNA content and related protein expression in L6 myotubes.** (**A**) Representative images for visualization of the mitochondrial morphology *in vitro*. Scale bar, 10 μm. (**B**) The mitochondrial mass was quantified by calculating the fluorescent intensity of MitoTracker Red. (**C**) Relative mtDNA content. (**D**) The levels of PGC-1α, TFAM, mfn2 and drp1 in the whole muscle lysates were measured by western blotting; GAPDH was used as a loading control. (**E**) The bar charts show quantification of the indicated proteins. (**F**) Representative images of the western blotting results for TFAM, mfn2 and drp1 in the mitochondrial subfractions; VDAC1 was used as a loading control. (**G**) The bar charts show quantification of the indicated proteins. Data are expressed as the mean ± SD. ^a^*P* < 0.05 vs. the control group; ^b^*P* < 0.05 vs. the PA-treated group; ^c^*P* < 0.05 vs. the RSV (25 μM) and PA (0.75 mM) cotreated group with control siRNA transfection.

For the morphological analysis of mitochondria, L6 myotubes were labeled with MitoTracker probes. As shown in Figure 7A, the mitochondria of the control group were filamentous and exhibited a thread-like appearance. PA treatment resulted in punctate and highly fragmented mitochondria, whereas RSV administration attenuated these abnormal morphological changes. A further analysis of the fluorescence intensity showed that RSV prevented the PA-induced reduction in mitochondrial mass (Figure 7B). However, PKA siRNA, LKB1 siRNA and AMPK siRNA transfection abrogated these effects of RSV (Figure 7A and 7B). Similar alterations were observed for the mtDNA content (Figure 7C).

We subsequently evaluated the effects of RSV on several mitochondrial-related protein levels. The results showed that both the decreases in the PGC-1α, TFAM and mfn2 protein levels and the increase in drp1 expression in PA-treated myotubes were rescued by RSV treatment (Figure 7D and 7E). Similar results were found for TFAM, mfn2 and drp1 protein expression in the mitochondrial subfractions (Figure 7F and 7G). However, PKA siRNA, LKB1 siRNA and AMPK siRNA transfection abolished these effects of RSV (Fig 7D-G). Together, these results indicated that RSV improved the mitochondrial morphology, restored the mtDNA copy number and reversed mitochondrial-related protein expression via the PKA/LKB1/AMPK signaling pathway *in vitro*.

### RSV reversed PA-induced mitochondrial dysfunction *in vitro*

**Figure 8:**
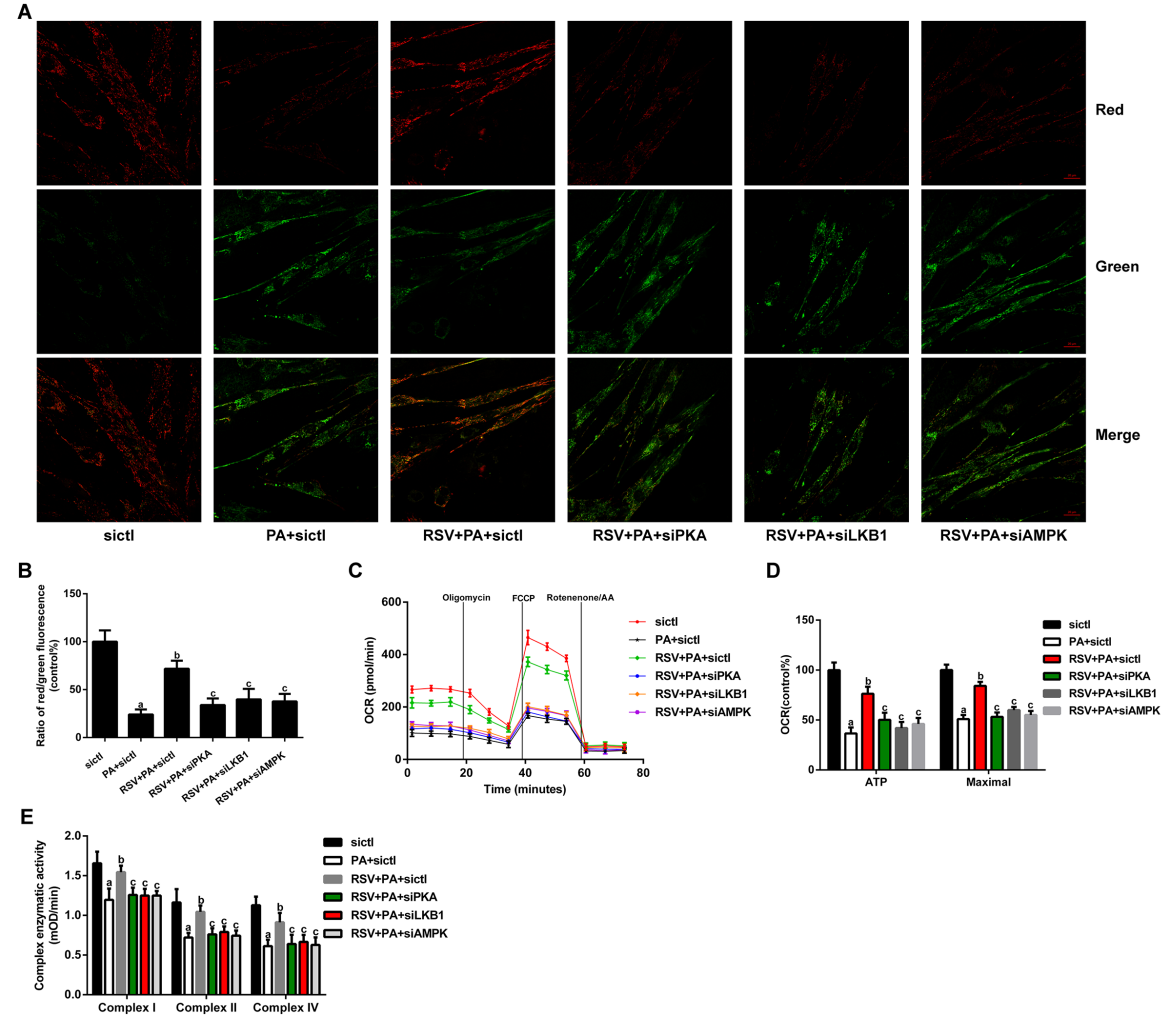
**Effects of RSV on mitochondrial function in L6 myotubes.** (**A**) Representative images used for determination of the Δψm *in vitro.* Scale bar, 20 μm. (**B**) Ratio of red to green fluorescence. (**C**) Respiration curve of each group. (**D**) The bar charts show the quantification of ATP production and maximal respiration. (**E**) Activities of complexes I, II, and IV. Data are expressed as the mean ± SD. ^a^*P* < 0.05 vs. the control group; ^b^*P* < 0.05 vs. the PA-treated group; ^c^*P* < 0.05 vs. the RSV (25 μM) and PA (0.75 mM) cotreated group with control siRNA transfection.

The Δψm was measured by JC-1 staining. As shown in Figure 8A and 8B, the ratio of red to green fluorescence was notably decreased after PA treatment, which suggested that the Δψm was depolarized. However, RSV administration prevented the PA-induced loss of Δψm. In addition, the protective effects of RSV on Δψm were abrogated by PKA siRNA, LKB1 siRNA and AMPK siRNA.

We then analyzed the oxygen consumption rate (OCR) in myotubes. The data showed that RSV treatment prevented the PA-induced declines in the mitochondrial respiration capacity, as indicated by the restoration of ATP production and maximal respiration (Figure 8C and 8D). However, PKA siRNA, LKB1 siRNA and AMPK siRNA transfection abolished these effects of RSV (Figure 8C and 8D). We subsequently measured the activities of mitochondrial respiratory chain complexes and found that RSV treatment restored the reduced activities of complexes I, II, and IV (Figure 8E). These effects of RSV were also reversed by PKA siRNA, LKB1 siRNA and AMPK siRNA (Figure 8E). Together, these results suggested that RSV attenuated PA-induced mitochondrial dysfunction via the PKA/LKB1/AMPK signaling pathway *in vitro.*

### RSV alleviated PA-induced oxidative stress *in vitro*

**Figure 9:**
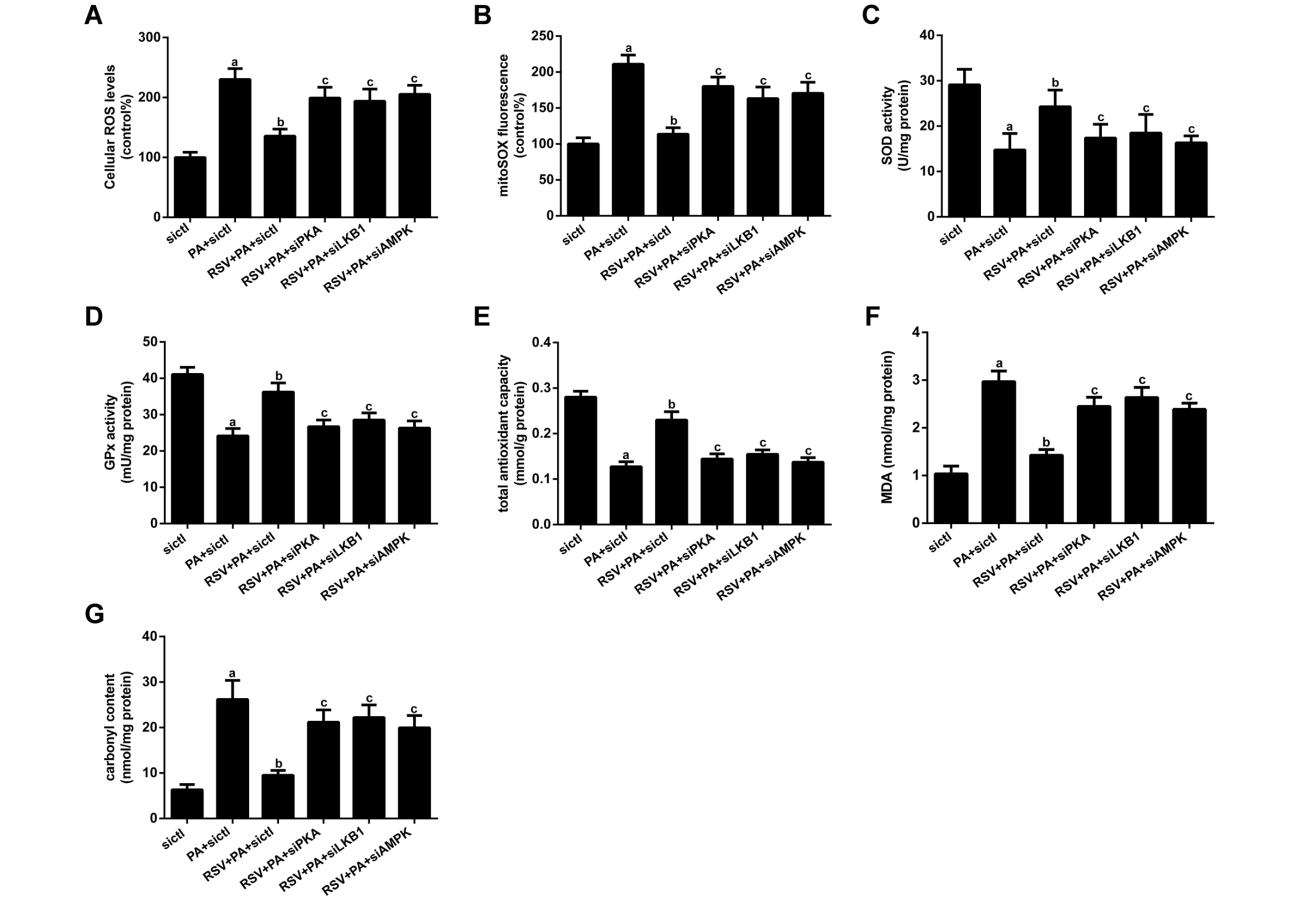
**Effects of RSV on oxidative stress in L6 myotubes.** (**A**) Cellular total ROS levels. (**B**) mtROS levels. (**C** and **D**) Activities of SOD and GPx *in vitro*. (**E**) T-AOC *in vitro.* (**F**) MDA levels. (**G**) Carbonyl protein content. Data are expressed as the mean ± SD. ^a^*P* < 0.05 vs. the control group; ^b^*P* < 0.05 vs. the PA-treated group; ^c^*P* < 0.05 vs. the RSV (25 μM) and PA (0.75 mM) cotreated group with control siRNA transfection.

We then investigated the effects of RSV on oxidative stress. As shown in Figure 9A and 9B, PA significantly elevated the cellular total reactive oxygen species (ROS) and mitochondrial ROS (mtROS) levels, which were inhibited by RSV treatment. Moreover, the decreases in T-AOC and the activities of SOD and GPx, as well as the increases in MDA and carbonyl protein levels in PA-treated myotubes, were all sufficiently normalized by RSV treatment (Fig 9C-G). However, PKA siRNA, LKB1 siRNA and AMPK siRNA transfection abrogated these effects of RSV (Figure 9). These results suggested that the protective effects of RSV on oxidative stress were exerted via the PKA/LKB1/AMPK signaling pathway *in vitro*.

### RSV improved protein metabolism via the PKA/LKB1/AMPK signaling pathway

**Figure 10:**
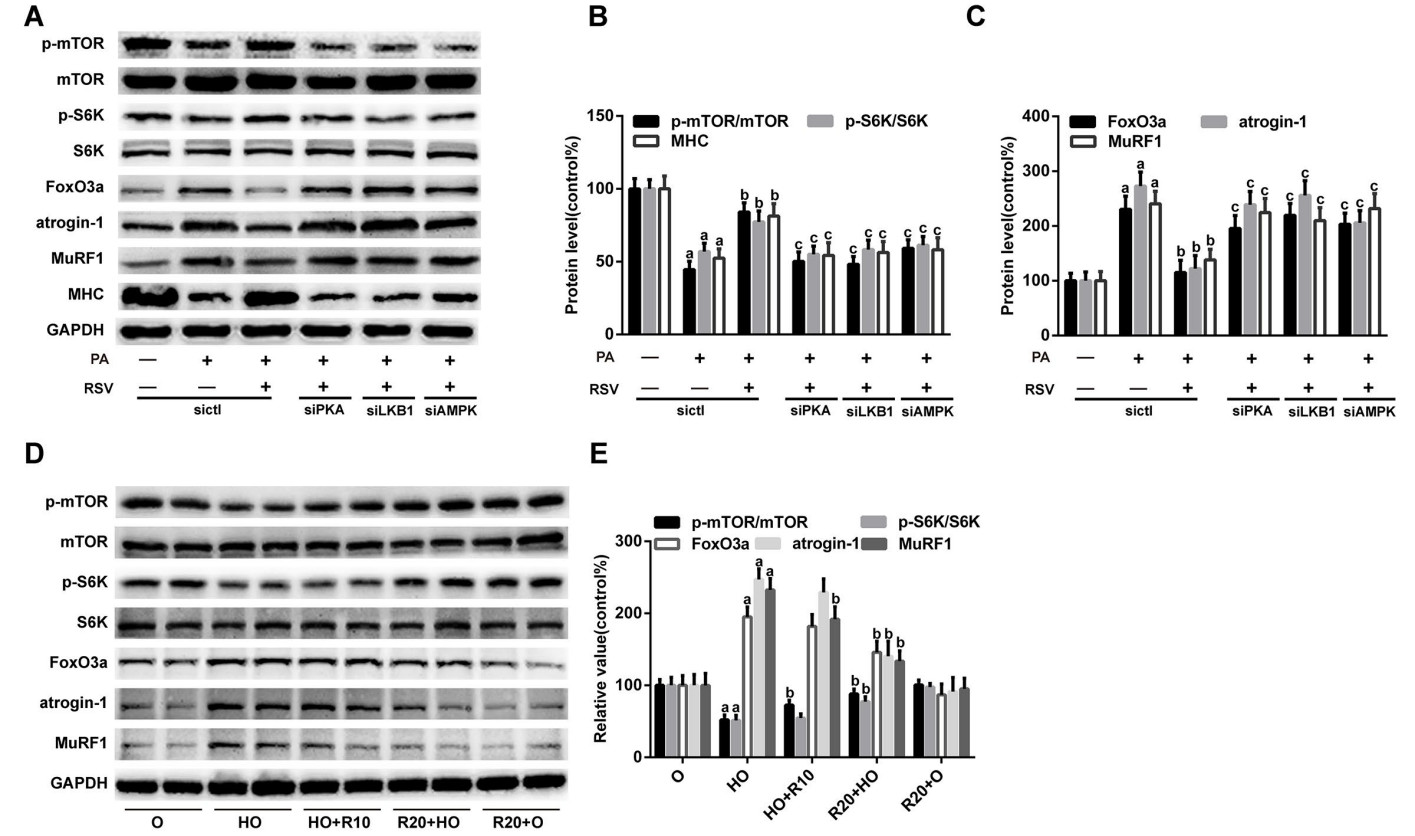
**RSV improves protein metabolism via the PKA/LKB1/AMPK signaling pathway.** (**A**) Representative images of the western blotting results for p-mTOR, mTOR, p-S6K, S6K, FoxO3a, atrogin-1, MuRF1 and MHC in L6 myotubes; GAPDH was used as a loading control. (**B** and **C**) The bar graphs show quantification of the indicated proteins. Data are expressed as the mean ± SD. ^a^*P* < 0.05 vs. the control group; ^b^*P* < 0.05 vs. the PA-treated group; ^c^*P* < 0.05 vs. the RSV (25 μM) and PA (0.75 mM) cotreated group with control siRNA transfection. (**D**) Representative images of the western blotting results for p-mTOR, mTOR, p-S6K, S6K, FoxO3a, atrogin-1 and MuRF1 in the GAS muscles; GAPDH was used as a loading control. (**E**) The bar charts show the relative protein levels. Data are expressed as the mean ± SD. ^a^*P* < 0.05 vs. the O group; ^b^*P* < 0.05 vs. the HO group.

Muscle atrophy occurs when the rate of protein degradation exceeds that of protein synthesis [33]. Thus, we further evaluated the expression of several key factors involved in protein metabolism. As shown in Fig 10A and 10C, the protein levels of FoxO3a, atrogin-1 and MuRF1, all of which are well-known markers of protein degradation and muscle atrophy, were robustly elevated by PA treatment but significantly inhibited by the administration of RSV *in vitro.* RSV also prevented the decline in protein synthesis, as evidenced by the restoration of p-mTOR and p-S6K expression in PA-treated myotubes (Figure 10A and 10B). Similar results were found in the GAS muscles of HFD-fed aged rats after RSV supplementation (Figure 10D and 10E). However, PKA siRNA, LKB1 siRNA and AMPK siRNA transfection abolished the effects of RSV on these protein metabolism-related factors *in vitro* (Figure 10A-C). In addition, the protective effects of RSV on the MHC content were also blocked by PKA siRNA, LKB1 siRNA and AMPK siRNA (Figure 10A and 10B). These results indicated that RSV improved protein metabolism and attenuated skeletal muscle atrophy through the PKA/LKB1/AMPK signaling pathway.

### RSV improved protein metabolism by reversing mitochondrial dysfunction and oxidative stress

**Figure 11:**
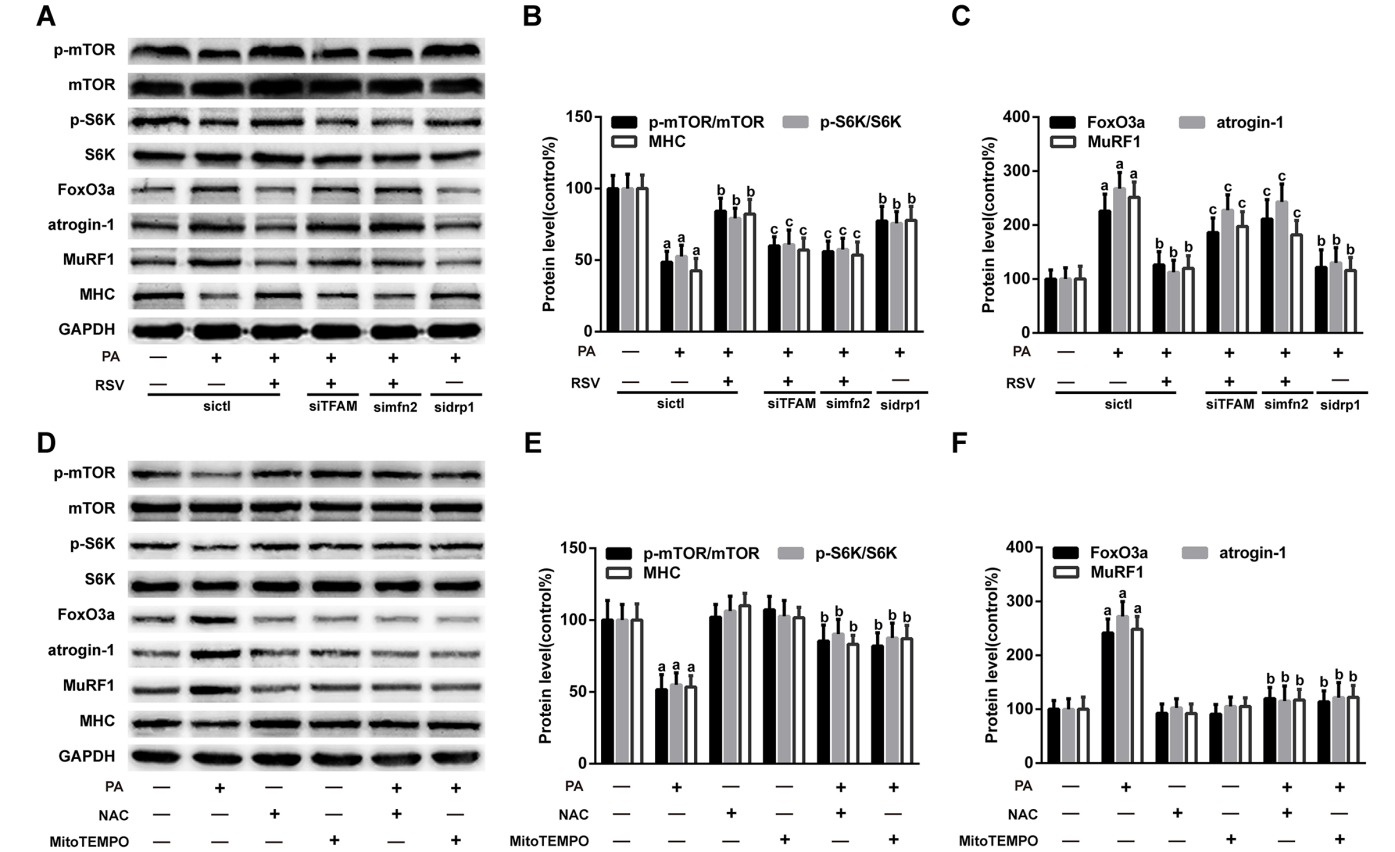
**RSV improves protein metabolism by improving mitochondrial function and oxidative stress.** (**A**) Representative images of the western blotting results for p-mTOR, mTOR, p-S6K, S6K, FoxO3a, atrogin-1, MuRF1 and MHC in L6 myotubes; GAPDH was used as a loading control. (**B** and **C**) The bar charts show quantification of the indicated proteins. Data are expressed as the mean ± SD. ^a^*P* < 0.05 vs. the control group; ^b^*P* < 0.05 vs. the PA-treated group; ^c^*P* < 0.05 vs. the RSV (25 μM) and PA (0.75 mM) cotreated group with control siRNA transfection. (**D**) Myotubes were incubated with PA for 24 h in the presence or absence of NAC (5 mM) or MitoTEMPOL (5 μM). Protein levels were measured by western blotting. (**E**) The bar charts show the relative protein levels. Data are expressed as the mean ± SD. ^a^*P* < 0.05 vs. the control group; ^b^*P* < 0.05 vs. the PA-treated group.

Previous studies have demonstrated that mitochondrial dysfunction decreases protein synthesis and increases protein degradation [10]. We therefore hypothesized that the RSV-mediated improvement in muscle protein metabolism was linked to its role in reversing mitochondrial dysfunction and oxidative stress through the PKA/LKB1/AMPK signaling pathway. To verify this hypothesis, we transfected L6 myotubes with TFAM siRNA, mfn2 siRNA and drp1 siRNA. As shown in Fig. S2B and S2C, TFAM siRNA and mfn2 siRNA transfection inhibited RSV-induced mitochondrial respiration recovery, and the transfection of these siRNAs also diminished the effects of RSV on the FoxO3a, atrogin-1, MuRF1, p-mTOR, p-S6K and MHC levels in myotubes (Figure 11A-C). In addition, drp1 siRNA transfection improved the mitochondrial respiration capacity and protein metabolism in PA-treated myotubes, and these effects were similar to those obtained with RSV (Figure 11A-C, S2B and S2C).

Oxidative stress has also been shown to inhibit protein synthesis and promote proteolysis in skeletal muscle [34]. To investigate the role of oxidative stress in muscle protein metabolism, the ROS scavenger N-acetyl-L-cysteine (NAC) and mitochondria-targeted antioxidant MitoTEMPO were used to suppress oxidative stress in myotubes. The results showed that both NAC and MitoTEMPO markedly decreased cellular ROS levels in PA-treated myotubes (Figure S2D). Furthermore, NAC and MitoTEMPO mimicked the effects of RSV on protein metabolism-related factors and MHC content (Figure 11D-F). Additionally, NAC and MitoTEMPO alone exerted no significant effects on the protein levels of FoxO3a, atrogin-1, MuRF1, p-mTOR, p-S6K and MHC (Figure 11D-F).

Based on these findings, we concluded that RSV improved protein metabolism and prevented HFD- and PA-induced muscle atrophy by reversing mitochondrial dysfunction and oxidative stress.

## DISCUSSION

Aging research has received great interest throughout human history. Because skeletal muscle is an important target of antiaging interventions [3], a number of studies have focused on muscle atrophy in older subjects. Growing evidence suggests that obesity and saturated fatty acids can augment sarcopenia [35–37], leading to the coexistence of reduced muscle mass and excess fat mass. This condition, which is defined as sarcopenic obesity, has become a critical public health challenge over recent years [7]. To date, there are no satisfactory strategies for its management [8]. Hence, the identification of novel pharmacological targets and the development of effective approaches to combat sarcopenic obesity have important functional implications for older adults.

RSV has been shown to attenuate muscle atrophy under several conditions [29]. However, little information is available regarding the effects of RSV on obesity-induced muscle atrophy in older adults. In the present study, aged rats were fed the HFD to induce sarcopenic obesity models. Our results showed that RSV administration inhibited muscle loss and the decrease in the average fiber CSA observed in the HFD-fed rats, and these effects contributed to the restoration of grip strength obtained with RSV. Moreover, previous studies have indicated that a reduction in the efficiency of ATP production will result in reduced myofiber oxidative capacity and skeletal muscle weakness [38–40]. Thus, the RSV-mediated improvement of muscle strength might also be associated with its role in preserving the ATP levels. To verify the findings obtained in rats, PA, one of the most increased plasma fatty acids in obesity, was used in myotubes to mimic HFD-induced muscle atrophy *in vivo*, and the results showed that RSV improved the protein metabolism and restored the decreased MHC content and myotube diameter in PA-treated myotubes. These findings provide new evidence regarding the protective effects of RSV on obesity and saturated fatty acid-induced muscle atrophy. We further explored the potential mechanisms involved.

Mitochondrial function plays an important role in the regulation of skeletal muscle remodeling [41]. Previous studies have shown that HFD and PA induce mitochondrial dysfunction in skeletal muscle cells [42–44]. Consistent with these observations, we found that mitochondrial function was impaired in both HFD-fed rats and PA-overloaded myotubes. RSV treatment repressed the collapse of Δψm, enhanced mitochondrial respiratory chain complex activities and increased ATP content in skeletal muscle, which suggested that RSV exerts protective effects on mitochondrial function. To characterize the mechanisms associated with the RSV-mediated improvements in mitochondrial function, we subsequently assessed mitochondrial biogenesis and dynamics.

Mitochondrial biogenesis is crucial for the maintenance of mitochondrial abundance and function [45], and RSV has been reported to stimulate mitochondrial biogenesis in skeletal muscle during diabetes and disuse-induced muscle atrophy [28,46]. In this study, the protein levels of PGC-1α and TFAM, which are key regulators of mitochondrial biogenesis, were decreased by HFD and PA treatment but were rescued by RSV administration. Moreover, RSV restored the mtDNA content and mitochondrial mass in muscle cells. These data indicated that RSV promoted mitochondrial biogenesis in HFD- and PA-induced muscle atrophy models.

Mitochondrial dynamics is important for the maintenance of mitochondrial homeostasis [47]. Mfn2, a mitochondrial fusion protein, is highly expressed in skeletal muscle [48]. Previous reports have suggested that the mfn2 protein levels are reduced in the skeletal muscle of obese rats and type 2 diabetic patients [49]. Moreover, a recent study by Sebastián and colleagues demonstrated that mfn2 deficiency in the skeletal muscle enhances age-induced mitochondrial dysfunction and promoted sarcopenia [50]. Drp1, a key mitochondrial fission protein, is critical for skeletal muscle mitochondrial maintenance [51]. Touvier et al. showed that the muscle-specific overexpression of drp1 led to mitochondrial damage and muscle mass loss [52]. Collectively, these findings indicate that abnormal changes in mfn2 and drp1 levels can impair the normal remodeling of the mitochondrial network and contribute to muscle loss. Our results showed that RSV treatment reversed the decreased mfn2 levels and increased drp1 levels both *in vivo* and *in vitro,* which suggested that RSV improved mitochondrial dynamics by promoting fusion and suppressing fission. In addition, it has been shown that PGC-1α controls mitochondrial dynamics by stimulating mfn2 expression [53], and drp1 and mfn2 are considered contributors to mtDNA integrity and distribution within the mitochondrial network [54,55]. Thus, RSV might improve mitochondrial morphology and function through the orchestration of mitochondrial biogenesis and mitochondrial dynamics.

Mitochondrial dysfunction is closely linked with oxidative stress, as mitochondria are both the major sources and primary targets of ROS [56]. Inappropriate electron leakage from mitochondrial respiratory chain complexes can lead to excessive mtROS formation [57]. Complementarily, antioxidant defense systems play a vital role in the maintenance of cellular redox homeostasis [58]. Our data showed that RSV protected against HFD- and PA-induced oxidative damage in skeletal muscle, and these effects were mediated through not only decreases in mtROS generation but also increases in antioxidant enzyme activities. In addition, given that RSV has free radical scavenging capacity [59], it is possible that RSV alleviates oxidative damage in skeletal muscle by directly scavenging excessive ROS.

The important roles of mitochondrial dysfunction and oxidative stress in inhibiting protein synthesis and promoting proteolysis in skeletal muscle have been revealed in previous studies [10,34]. Our results demonstrated that the RSV-mediated improvement in muscle protein metabolism was closely associated with its role in reversing mitochondrial dysfunction and oxidative stress. Moreover, FoxO3a and mTOR have been shown to regulate mitochondrial function [60–62], and an imbalanced protein metabolism can in turn increase or decrease the levels of normal mitochondrial proteins and thereby exacerbate mitochondrial damage. Thus, RSV might prevent muscle atrophy by coordinating the interaction between mitochondrial function and protein metabolism. In addition, the oxidative modification of muscle proteins can enhance their susceptibility to proteolysis and lead to further cellular damage or dysfunction [63]. Therefore, the RSV-mediated decrease in the carbonyl protein levels might also contribute to the anti-muscle atrophy effects of RSV.

To investigate the intervention time-related effects of RSV, we compared the effects of RSV on the muscles of the rats in the R20+HO and the HO+R10 groups. Although no significant difference in the plasma RSV content were found between these two groups, the rats administered RSV for 20 weeks showed significant increases in the GAS muscle weight and grip strength compared with the rats administered RSV for 10 weeks. These findings suggested that longer-term or early supplementation with RSV exerted a greater protective effect on HFD-induced muscle atrophy in aged rats. Moreover, we found that supplementation with RSV for 20 weeks exerted greater protective effects on HFD-induced mitochondrial dysfunction and oxidative stress in aged rats, which might account for the superior effects of longer-term RSV supplementation on sarcopenic obesity.

The effects of RSV on age-related muscle loss were also investigated, and we found that RSV alleviated aging-induced oxidative damage in skeletal muscle but had no significant effects on sarcopenia, which was consistent with previous findings by Jackson [31]. However, our results showed that the age-related decrease in muscle force was not alleviated by RSV, which was in contrast to the recent report by Liao and colleagues [64]. The differing results might be due to differences in the timing and dosage of RSV treatment. Previous studies have shown that the maintenance of an advantageous oxidative status might facilitate an extended healthy life span [65]. Thus, the ability of RSV to improve oxidative stress in the skeletal muscle of aged rats might contribute to its antiaging effects.

We further evaluated the mechanisms through which RSV improved mitochondrial function and oxidative stress and thereby prevented HFD- and PA-induced muscle atrophy. AMPK, a potential longevity target [66], plays an important role in the regulation of skeletal muscle mass and regeneration [67]. Previous studies have demonstrated that AMPK activation prevents inflammation and angiotensin II-induced muscle wasting [68,69]. Moreover, the activation of AMPK has also been reported to improve muscle pathology in Duchenne muscular dystrophy and spinal muscular atrophy models [70,71]. Furthermore, Bujak et al. found that the genetic deletion of skeletal muscle AMPK accelerates aging-induced myopathy and mitochondrial dysfunction [72]. However, other reports have indicated that AMPK activation enhances ubiquitin-proteasome-mediated catabolism in skeletal muscle [73,74]. These inconsistent findings might be due to differences in muscle wasting conditions. RSV has been shown to activate AMPK in skeletal muscle both *in vivo* and *in vitro* [75,76]. However, a previous study showed that RSV attenuates dexamethasone-induced muscle atrophy through inhibition of the AMPK pathway [77]. In this study, we found that HFD and PA induced a substantial decrease in the p-AMPK/AMPK ratio in skeletal muscle, whereas RSV treatment prevented this change. Moreover, AMPK siRNA transfection abolished the protective effects of RSV on mitochondrial function and oxidative stress. Furthermore, both the increases in MHC, p-mTOR and p-S6K expression and the decreases in FoxO3a, atrogin-1 and MuRF1 expression induced by RSV were reversed by AMPK siRNA. These results indicated that the beneficial effects afforded by RSV administration might occur through the activation of AMPK.

The signal molecule upstream of AMPK was examined. A previous study provided abundant evidence showing that LKB1 is the primary protein kinase acting upstream of AMPK [78]. Our results showed that LKB1 siRNA transfection inhibited the RSV-mediated activation of AMPK in myotubes, and the RSV-induced improvements in mitochondrial function, oxidative stress and protein metabolism were all abrogated by LKB1 siRNA. Although the LKB1/AMPK pathway has been found to be responsible for the beneficial effects of RSV on endothelial cells and cardiac myocytes [79,80], our results provide the first evidence demonstrating the crucial role of this pathway in RSV-induced protection against mitochondrial dysfunction and oxidative stress in skeletal muscle, and these findings might provide new insights into the anti-muscle atrophy effects of RSV.

LKB1 can be activated through phosphorylation at Ser431 by PKA [22]. Thus, the possible involvement of the PKA/LKB1/AMPK pathway in the RSV-induced protective effects on muscle atrophy was also investigated. As expected, RSV triggered PKA phosphorylation and activation, which are required for activation of the LKB1/AMPK pathway. These findings indicated that the PKA/LKB1/AMPK pathway was involved in the RSV-induced improvements in mitochondrial function and oxidative stress and the subsequent counteraction of HFD- and PA-induced muscle atrophy.

Previous studies have shown that obesity and sarcopenia are strongly interconnected and can be reciprocally regulated through complex mechanisms [81]. The vicious cycle between the accumulation of excessive fat mass and the loss of skeletal muscle mass can further impair the quality of life of elderly individuals. In addition, it has been found that fat infiltration into muscle can augment the muscle atrophy process [82]. Thus, the reduction of body and intramuscular fat might also be an effective approach for the management of sarcopenic obesity. Our results showed that RSV supplementation prevented HFD-induced increased body fat mass and intramuscular fat deposition and thereby contributed to the inhibition of muscle loss. Thus, RSV might prevent HFD-induced muscle atrophy in aged rats independent of its effects on mitochondrial function and oxidative stress in skeletal muscle. Further studies are warranted to explore the mechanisms responsible for the effects of RSV on fat accumulation in HFD-induced sarcopenic obesity models.

In summary, we demonstrated that RSV prevented HFD-induced muscle atrophy in aged rats by improving mitochondrial function and oxidative stress through the PKA/LKB1/AMPK pathway. These findings provide evidence showing the potential protective effects of RSV on sarcopenic obesity and might have important theoretical and application prospects for aging research.

## MATERIALS AND METHODS

### Reagents and antibodies

RSV, PA, Protein Carbonyl Content Assay Kit, NAC and MitoTEMPO were purchased from Sigma-Aldrich (St. Louis, MO, USA). Cell Counting Kit-8 (CCK-8) was obtained from Dojindo Laboratories (Kumamoto, Japan), and glass-bottom cell culture dishes were obtained from Nest Biotechnology (Wuxi, China). MitoTracker Deep Red and MitoSOX Red were obtained from Invitrogen (Carlsbad, CA, USA). Antibodies against MHC (ab 24642), PGC-1α (ab 106814), TFAM (ab 131607), mfn2 (ab 50838), FoxO3a (ab 23683) and VDAC1 (ab 15895) were purchased from Abcam (Cambridge, UK). Antibodies against LKB1 (3047), p-LKB1 (3482), AMPK (2532), p-AMPK (2531), p-mTOR (2971), mTOR (2972), p-S6K (9205), S6K (9202) and GAPDH (5174) were purchased from Cell Signaling Technology (Beverly, MA, USA). Antibodies against PKA (sc-903), p-PKA (sc-12905), atrogin-1 (sc-33782) and MuRF1 (sc-27642) were obtained from Santa Cruz Biotechnology (Santa Cruz, CA, USA). The antibody against drp1 (BD611113) was obtained from BD Biosciences (San Jose, CA, USA).

### Animals

Young (aged 3 months) and old (aged 18 months) male Sprague-Dawley (SD) rats were obtained from the animal center of the Third Military Medical University (Chongqing, China). The rats were maintained in collective cages and housed under pathogen-free conditions with constant temperature and humidity, a regular 12-h light:12-h dark cycle and free access to water and food. All the animal experiments were performed in accordance with the institutional guidelines on animal experimentation set by the National Institutes of Health and were approved by the Institutional Animal Care and Use Committee of the Third Military Medical University.

### Diets and experimental design

The rats were fed either a CD or a HFD for 20 weeks. The CD contained 10% kcal from fat, whereas the HFD contained 45% kcal from fat. RSV was added to the diet as described previously [83]. First, 50 g powdered CD or HFD was weighed and well mixed with 55 mL double-distilled H2O. Next, 15 mL ethanol containing 0.2 g RSV (for 0.4% in the diet) was thoroughly mixed with the diet, and the pellets were then reconstituted. The same volume of ethanol was added to the control diet. For ethanol evaporation, the diet was placed in a vacuum oven at 50 °C overnight. All the diets were irradiated by gamma irradiation and stored in light-protected and airtight containers at 4 °C.

The rats were randomly divided into the following six groups (n=10 per group): young rats fed the CD (Y), old rats fed the CD (O), old rats fed the HFD (HO), old rats fed the HFD for 10 weeks and then the HFD supplemented with RSV for 10 weeks (HO+R10), old rats fed the HFD supplemented with RSV starting from the time of study initiation and throughout the experimental period (R20+HO), and old rats fed the CD supplemented with RSV starting from the time of study initiation and throughout the experimental period (R20+O). Body weight was measured weekly, and food intake was recorded every 2–3 days. Grip strength was determined using a grip strength meter (YuYan Instruments, Shanghai, China).

At the end of the 20-week study period, the rats were fasted overnight and then anesthetized with sodium pentobarbital anesthesia. All efforts were made to alleviate suffering. Fresh blood samples, muscle tissues and adipose tissues were harvested for further analyses.

### Serum parameter analysis

Serum was separated by solidification and centrifugation (4 °C, 3,000×g, 10 min). The levels of FFA, TG, TC, HDL-C, and LDL-C in serum were measured using an automatic analyzer (Olympus AU5400, Japan). The concentrations of RSV in serum were measured by liquid chromatography-mass spectrometry according to our previous studies [84].

### Histological analysis

Fresh muscle tissues were fixed in 4% paraformaldehyde, embedded in paraffin wax, transversely sectioned into 5-μm slices, and stained with hematoxylin and eosin (H&E) following standard protocols. Photomicrographs were captured using an Olympus VS120 microscope. The myofiber CSA was measured using ImageJ software (NIH) by a researcher blinded to the experimental groups.

### Body fat measurement

The body fat volume was analyzed using a Quantum FX MicroCT Imaging System (Perkin Elmer Inc., Waltham, MA, USA). The rats were anesthetized with 5% chloral hydrate (4 ml/kg body weight) and then subjected to the microCT scans. The images were acquired from the abdominal region (between vertebrae L1 and L6) of the rats. To vividly observe adipose tissues, two-dimensional grayscale image slices were reconstructed into three-dimensional images. The fat volume was quantitatively analyzed using Analyze 12.0 software (Analyze Direct, Overland Park, KS, USA) in a blinded manner.

### Cell culture and treatments

The rat L6 myogenic cell line (obtained from ATCC-CRL-1458, Manassas, VA, USA) was grown in Dulbecco’s-modified Eagle’s medium (DMEM; Gibco-Invitrogen, Carlsbad, CA, USA) containing 10% fetal bovine serum (FBS; HyClone, USA) and 1% penicillin/streptomycin in a humidified incubator enriched with 5% CO_2_ at 37 °C. The medium was then replaced with DMEM containing 2% horse serum (Gibco, New Zealand) for the induction of myotube formation. After differentiation, the cells were treated with PA in the presence or absence of RSV for 24 h.

### Cell viability measurement

Cell viability was analyzed through the CCK-8 assay according to the manufacturer’s instructions. Briefly, the cells were seeded into 96-well microplates at a density of 8,000 cells/well and differentiated into myotubes. After exposure to the indicated treatments, CCK-8 solution (20 μL/well) was added to each well, and the microplate was incubated at 37 °C for 2 h. Cell viability was determined through absorbance measurements using an Infinite**™** M200 Microplate Reader (Tecan, Mannedorf, Switzerland) at 450 nm. The results are expressed as percentages relative to the control group values.

### siRNA transfection

siRNAs targeting PKA, LKB1, AMPK, TFAM, mfn2, and drp1, as well as control siRNA, were obtained from Santa Cruz Biotechnology. Myotubes were transfected with 100 nM siRNA for 6 h according to the manufacturer’s directions. The cells were then transferred to fresh medium and incubated for an additional 24 h. Where indicated, the cells were treated with RSV and PA for 24 h. Thereafter, cells were harvested for subsequent experiments.

### Measurement of the myotube diameter

To analyze the myotube diameter, images were captured under a phase contrast microscope at 100× magnification. The diameters of a total of 60 myotubes from at least 10 random fields were measured using ImageJ software (NIH) by a researcher blinded to the group designations. The results are expressed as percentages relative to the control group value.

### Measurement of the TG content and ATP levels

The TG levels of the GAS muscles and myotubes were determined using a TG assay kit (Nanjing Jiancheng Biotechnology, China) following the manufacturer’s protocol. The protein concentrations were measured with a BCA Protein Assay Kit (Beyotime, China). The TG content is expressed as mmol/g protein. The ATP levels in the GAS muscles were measured using an ATP Assay Kit (Beyotime, China) according to the manufacturer’s instructions.

### Mitochondrial morphology analysis

The changes in the mitochondrial morphology of the GAS muscles were observed by TEM. Immediately after the rats were sacrificed, the GAS muscles were dissected, trimmed to approximately 1.0-mm^3^ pieces, fixed in 2.5% glutaraldehyde solution and subsequently in 1% osmium tetroxide, dehydrated, and then embedded in epoxy resin. The tissues were then cut using an RMC/MTX ultramicrotome (Elexience). Ultrathin sections (60 nm) stained with 2% uranyl acetate and lead citrate were observed under a JEM-1400 microscope (JEOL, Japan) at 25,000× magnification.

For visualization of the mitochondrial network in L6 myotubes, the cells were cultured in a cell culture dish, subjected to the indicated treatment, loaded with 250 nM MitoTracker Deep Red probe and incubated for 30 min at 37 °C. The cells were then gently washed with prewarmed PBS and observed under a Zeiss confocal laser scanning microscope (Carl Zeiss, LSM 800). The mitochondrial fluorescence intensity was quantified using Zeiss LSM Image Examiner software. The results were obtained from at least 30 cells in each group from three experiments.

### Determination of the Δψm

The Δψm in the GAS muscles and myotubes was measured using a JC-1 probe (Beyotime, China). For muscles, the freshly isolated mitochondria were suspended in JC-1 staining solution, and the fluorescence intensity was detected using an Infinite**™** M200 Microplate Reader. For myotubes, the cells were grown in a cell culture dish, subjected to the indicated treatment, incubated with JC-1 staining solution for 20 min, gently washed with PBS and resuspended in serum-free medium. Images were acquired using a Zeiss confocal laser scanning microscope, and further analysis of the fluorescence intensity was performed using Zeiss LSM Image Examiner software. The Δψm is represented by the ratio of red to green fluorescence.

### mtDNA quantification

mtDNA was isolated from GAS muscles and myotubes using a Mito DNA Extraction Kit (Genmed Scientifics, Inc., USA) following the manufacturer’s protocol. DNA was quantified spectrophotometrically (260 nm) and subjected to quantitative real-time PCR using a qTower 2.2 real-time PCR system (Analytik Jena, German). The relative mtDNA content was obtained by comparing the amplification products to those of ACTB (forward: 5'- CCACCATGTACCCAGGCATT-3'; reverse: 5'-CGGACTCATCGTACTCCTGC-3') and NADH-CoQ oxidoreductase 1 (forward: 5'-TTAATTGCCATGGC CTTCCTCACC-3'; reverse: 5'-TGGTTAGAGGGCG TATGGGTTCTT-3').

### Measurement of the cell mitochondrial OCR

L6 myoblasts were seeded into XF Cell Culture Microplates and differentiated into myotubes. After the indicated treatment, the medium was replaced with unbuffered XF Base Medium containing 1 mM pyruvate, 2 mM glutamine and 10 mM glucose. The microplates were then placed into a non-CO_2_ incubator for 1 h at 37 °C. Thereafter, oxygen consumption was measured using an Extracellular Flux Analyzer (Seahorse Bioscience, MA, USA) as previously described. Every point represents average data from five different wells, and the parameter was analyzed using Wave and Report Generator software.

### Mitochondrial complex activity assay

Mitochondria were isolated from GAS muscles or myotubes using Mitochondria Isolation Kits (Beyotime, China) according to the manufacturer’s instructions. The activities of complexes I, II, and IV were determined in freshly isolated mitochondrial homogenates using commercial assay kits from Abcam (Cambridge, MA, USA) following the manufacturer’s protocol.

### Antioxidant activity assay

The antioxidant capacity in the GAS muscles and myotubes was determined by measuring the T-AOC and activity of SOD and GPx using corresponding assay kits (Beyotime, China) according to the manufacturer’s instructions.

### Assessment of the ROS levels and mitochondrial superoxide (O_2_^•−^) levels in L6 myotubes

The total ROS levels in myotubes were measured using an ROS Assay Kit (Beyotime, China) following the manufacturer’s protocol. The mtROS levels were determined using MitoSOX Red, a highly selective fluorescent probe for the detection of mitochondrial O2•−. Briefly, the cells were seeded into a 24-well microplate. After differentiation and treatment, the myotubes were incubated with 5 μM MitoSOX Red reagent working solution in the dark for 10 min at 37 °C. After gentle washing, the fluorescence intensity was analyzed using an Infinite**™** M200 Microplate Reader.

### Measurement of the MDA levels and carbonyl protein content

The MDA content was determined as a marker of lipid peroxidation in the GAS muscles and myotubes using an MDA Detection Kit (Beyotime, China) following the manufacturer’s instructions. The carbonyl protein level was measured as an indicator of protein oxidative damage in the GAS muscles and myotubes using a Protein Carbonyl Content Assay Kit according to the manufacturer’s protocol. The MDA levels and carbonyl protein content are expressed as nmol/mg protein.

### Western blotting

Total proteins from the GAS muscles were extracted using a Tissue Protein Extraction Solution (Invitrogen, USA) containing a protease inhibitor (Roche Applied Science, Germany) and a PhosSTOP Phosphatase inhibitor cocktail tablet (Roche Applied Science, Germany). Total cell lysates from L6 myotubes were obtained by homogenizing the cells in RIPA lysis buffer (Beyotime, China) supplemented with phenylmethanesulfonyl fluoride (PMSF; Beyotime, China) and a PhosSTOP phosphatase inhibitor cocktail tablet. For the preparation of mitochondrial proteins, the mitochondria isolated from the muscle tissues and myotubes were homogenized in mitochondrial lysis buffer containing PMSF. All the lysates were centrifuged at 14,000×g and 4 °C for 20 min. The supernatants were collected, and the protein concentrations were determined with a BCA Protein Assay Kit. The protein samples were then applied to SDS-PAGE gels, electrotransferred to PVDF membranes (Bio-Rad), blocked with 5% nonfat milk or 5% BSA in TBST for 2 h, incubated with primary antibodies at 4 °C overnight and then incubated with anti-rabbit or anti-mouse secondary antibodies for 1 h. The blots were visualized using an enhanced chemiluminescence system (Millipore, Billerica, MA, USA), and the densitometric analysis was performed using Quantity One software (Bio-Rad Laboratories, Hercules, CA, USA).

### Statistical analysis

The data analyses were performed using SPSS 19.0 software (Chicago, IL, USA). The quantitative data are presented as the mean ± standard deviations (SD). The statistical analyses were conducted using one-way analysis of variance (ANOVA) followed by Tukey–Kramer post hoc tests for multiple comparisons. The results were considered to be statistically significant if *P* < 0.05. Each experiment was performed a minimum of three times.

## SUPPLEMENTARY MATERIAL

Supplementary Figures
