# Supplementary material for: Resveratrol prevents sarcopenic obesity by reversing mitochondrial dysfunction and oxidative stress via the PKA/LKB1/AMPK pathway
Source: Aging (Albany NY). 2019 Apr 15;11(8):2217–40. doi: 10.18632/aging.101910 (PMC6519996; doi:10.18632/aging.101910)
Supplement: Supplementary Figures [file aging-11-101910-s001.pdf]

SUPPLEMENTARY MATERIAL

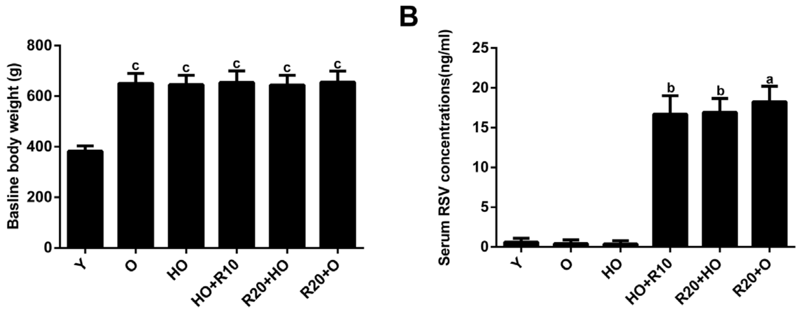

Figure S1. The baseline body weight and serum RSV concentrations. (**A**) The baseline body weight in each group. (**B**) Serum RSV levels. Data are expressed as the mean ± SD. [a]$P < 0.05$ vs. the O group; [b]$P < 0.05$ vs. the HO group; [c]$P < 0.05$ vs. the Y group.

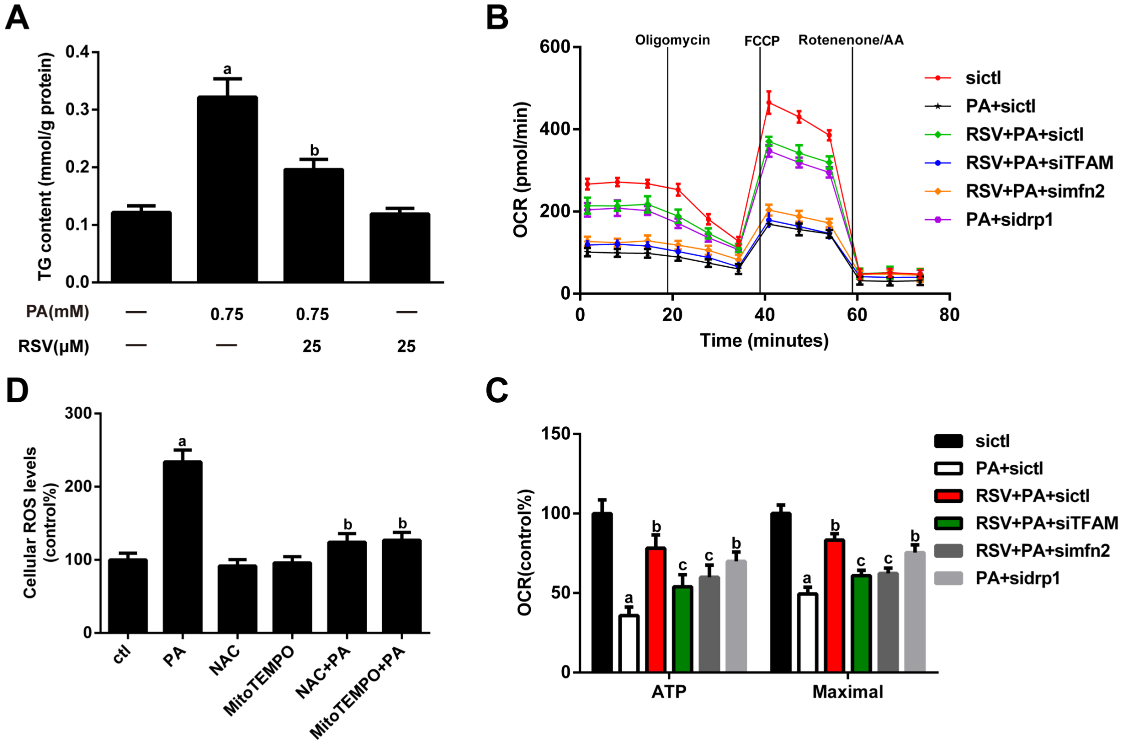

Figure S2. TG content, OCR and total ROS levels in L6 myotubes. (**A**) Intracellular TG content. (**B**) The respiration curve in each group. (**C**) The bar graphs show the quantification of ATP production and maximal respiration. (**D**) Cellular total ROS levels. Data are expressed as the mean ± SD. [a]$P < 0.05$ vs. the control group; [b]$P < 0.05$ vs. the PA-treated group; [c]$P < 0.05$ vs. the RSV (25 μM) and PA (0.75 mM) cotreated group with control siRNA transfection.
